# Effect of far-infrared radiation on inhibition of colonies on packaging during storage of sterilised surgical instruments

**DOI:** 10.1038/s41598-023-35352-9

**Published:** 2023-05-25

**Authors:** Li-Yun Fann, Chih-Chien Cheng, Yung-Chen Chien, Cheng-Wei Hsu, Wu-Chien Chien, Yao-Ching Huang, Ren-Jei Chung, Shi-Hao Huang, Ying-Hua Jiang, Shih-Han Yin, Kai-Wen Cheng, Yi-Ping Wu, Sheng-Huang Hsiao, Shao-Yuan Hsu, Ying-Che Huang, Chi-Ming Chu

**Affiliations:** 1 Department of Nursing, Taipei City Hospital, Taipei, 10684 Taiwan; 2 Department of Nurse-Midwifery and Women Health, National Taipei University of Nursing and Health Sciences, Taipei, 11220 Taiwan; 3 School of Public Health, National Defense Medical Center, Taipei, 11490 Taiwan; 4 Univeraity of Taipei, Taipei, 10048 Taiwan; 5 Department of Obstetrics/Gynecology, Taipei City Hospital, Taipei, 10341 Taiwan; 6 School of Medicine, College of Medicine, Fu Jen Catholic University, New Taipei City, 242062 Taiwan; 7 Department of Inspection, Taipei City Hospital, Ren-Ai Branch, Taipei, 10629 Taiwan; 8 Graduate Institute of Life Sciences, National Defense Medical Center, Taipei, 11490 Taiwan; 9 Department of Medical Research, Tri-Service General Hospital, Taipei, 11490 Taiwan; 10 Department of Chemical Engineering and Biotechnology, National Taipei University of Technology (Taipei Tech), Taipei, 10608 Taiwan; 11 Department of Neurosurgery, Taipei City Hospital, Ren-Ai Branch, Taipei, 10629 Taiwan; 12 Department of Anesthesia and Critical Care Medicine, Taipei City Hospital, Ren-Ai Branch, Taipei, 10629 Taiwan

**Keywords:** Biological techniques, Biotechnology

## Abstract

The sterilisation of surgical instruments is a major factor in infection control in the operating room (OR). All items used in the OR must be sterile for patient safety. Therefore, the present study evaluated the effect of far-infrared radiation (FIR) on the inhibition of colonies on packaging surface during the long-term storage of sterilised surgical instruments. From September 2021 to July 2022, 68.2% of 85 packages without FIR treatment showed microbial growth after incubation at 35 °C for 30 days and at room temperature for 5 days. A total of 34 bacterial species were identified, with the number of colonies increasing over time. In total, 130 colony-forming units were observed. The main microorganisms detected were *Staphylococcus* spp. (35%) and *Bacillus* spp. (21%) , *Kocuria marina* and *Lactobacillus* spp. (14%), and mould (5%). No colonies were found in 72 packages treated with FIR in the OR. Even after sterilisation, microbial growth can occur due to movement of the packages by staff, sweeping of floors, lack of high-efficiency particulate air filtration, high humidity, and inadequate hand hygiene. Thus, safe and simple far-infrared devices that allow continuous disinfection for storage spaces, as well as temperature and humidity control, help to reduce microorganisms in the OR.

## Introduction

The sterilisation cycle of surgical instruments is a major factor in infection control in the operating room (OR), and all items must be sterile to use for optimal patient safety^1^. Trays that are not regularly used must be frequently re-sterilised based on the surgical routine to ensure that they are sterile when the package is opened. The recommendation for the shelf life of sterile surgical packages is not based on any scientific evidence in most hospitals. Although sterilisation between uses is important to prevent the spread of microorganisms, it is also critical to properly handle the storage of sterilised packages to avoid contamination before the item is opened. According to the literature, microorganisms invade single-wrap muslin stored on open shelves as early as 3 days and double-wrap muslin in 21–28 days^2^. It is suggested that despite event-related shelf life, such as double-linen wrapping of stored sterile instruments, items must be re-sterilised every 2 weeks^3^, as microorganisms can contaminate the items in 28 days^4^. However, the packed items are regarded to be safe if used within 2 weeks^5^. In our OR, stored sterile items packed with double-wrapped cotton are used within 6 days. Frequent sterilisation requires frequent consumption of resources, such as labour, disinfection material, and time.

Steam sterilisation is the most commonly used method for sterilising surgical instruments worldwide^6^. The reprocessing cycle of surgical instruments comprises one of several processes, storage of sterilised items, recommended relative humidity of 40%–50%, and an ambient temperature of 15 °C–25 °C in a sterile storage area^7^. The admissible sterility assurance level of surgical instruments, which is the probability of obtaining a non-sterile item after a reprocessing cycle, is one in a million^8^; the risk increases if the procedures are not followed, thus increasing the transmission of pathogens via person-to-person or environmental factors. The presence of microorganisms in the OR is linked to several factors, such as the presence and activities of persons, temperature, and humidity, which are the main reasons for surgical-site infections after surgery^9–14^. Even after routine disinfection and sterilisation of the OR equipment, bacterial concentration was higher than that recommended by the CDC (30 colony-forming units [CFU]/m^3^) in 41% of the OR^15^. The movement of the surgical staff during operations can significantly increase the number of airborne particles by 8–31 times^16^. Surgical-site contamination is primarily attributable to airborne particles that may carry microorganisms^17^, which land on sterile instruments and the operator’s hands and are then transferred to the surgical site^18^. As a result, the movement of the staff can disrupt the laminar flow in the OR, resulting in increased contamination around the site. Considering the major role of air pollution, the dynamic quality of the OR air deserves close attention.

Ultraviolet light is the most frequently used method of environmental decontamination in healthcare settings, and there is evidence that far-infrared radiation (FIR) has germicidal properties^19,20^. Traditional sources of FIR are electric heaters^21^, and FIR exposure causes water molecules in the matrix of organisms to vibrate, generating heat that destroys microbial nucleic acids, proteins, and cell walls^22^. The literature on the biological effects and medical applications of FIR pointed out that FIR can inhibit microbial growth and reduce humidity^23^. FIR (λ = 3–100 μm) is in the electromagnetic spectrum A subdivision, which has been studied for its biological effects^24^, and it has been established that special lamps and saunas of FIR are safe, effective, and widely used sources of therapeutic effects^25^. Studies have also shown that the higher the temperature (300 °C), the faster the FIR achieves antibacterial effects and reduces humidity^26^. The microbial diversity of red snapper fillets after far-infrared thawing was significantly reduced^27^, and far-infrared ceramic beads of far-infrared sand bath therapy have bactericidal effects against *Micrococcus* spp., *Staphylococcus epidermidis*, *Bacillus cereus*, and non-glucose-fermentable Gram-negative *bacilli* on the skin^28^.

Studies have confirmed that the fabric used to package surgical instruments can retain more microorganisms after 20 washing processes and 20 cycles of autoclaving under real hospital conditions. However, it was also found that penetration by microorganisms did not occur in all samples, indicating that microorganisms could not enter the inner layer of the fabric^29^. In recent years, fibres impregnated with far-infrared-radiating ceramic nanoparticles and those woven into fabrics have been used as clothing and wraps^30,31^. Additionally, 100% cotton single-layer knitted fabric has good resistance to *Escherichia coli* and *S. aureus*^32^.

The aforementioned evidence suggests that while the double-layer cotton-wrapped instrument trays that have been autoclaved can still block microbial infiltration for at least 20 weeks, the autoclaving process itself will accelerate roughening of the cloth surface, increasing the probability of bacteria remaining on the cloth surface. When the cloth surface of the sterile packaging is contaminated with bacteria and then moved to the OR for use, it is easy for the microorganisms to transfer into air particles and spread in the OR, increasing the risk of surgical-site infections. Based on the ability of FIR to inhibit the growth of microorganisms and reduce humidity, this study aimed to determine the microbial distribution of cotton-packed equipment exposed to FIR and stored for a long time after steam sterilisation.

## Materials and methods

### Far-infrared equipment

**Figure 1:**
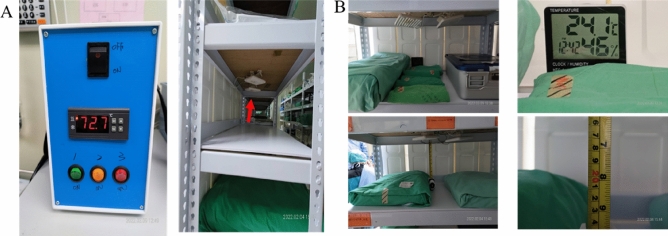
Far-infrared equipment. (**A**) Heat-able far-infrared device with a planar electrodynamic ceramic emitter with a maximum temperature of 80 °C. (**B**) The device was set up on the underside of the top shelf, and the vertical distance from the package surface is 20–22 cm.

We used a custom, heat-able, far-infrared device with a planar electrodynamic ceramic emitter with a maximum surface heating temperature of 80 °C and a wavelength of 2–22 μm at room temperature^28^ with a far-infrared radiated emissivity of 87% (Supplementary Fig. 1). The FIR equipment was set up on the top of the shelf, and the vertical distance from the bottom of the equipment bag surface was approximately 20–22 cm^33^. The timer device controlled the heating every 7 h and then shut down and cooled down to room temperature for 1 h. Subsequently, the timer device automatically turned on and reheated the far-infrared device. Three such consecutive cycles were performed per day (Fig. 1).

### Sampling procedure

**Figure 2:**
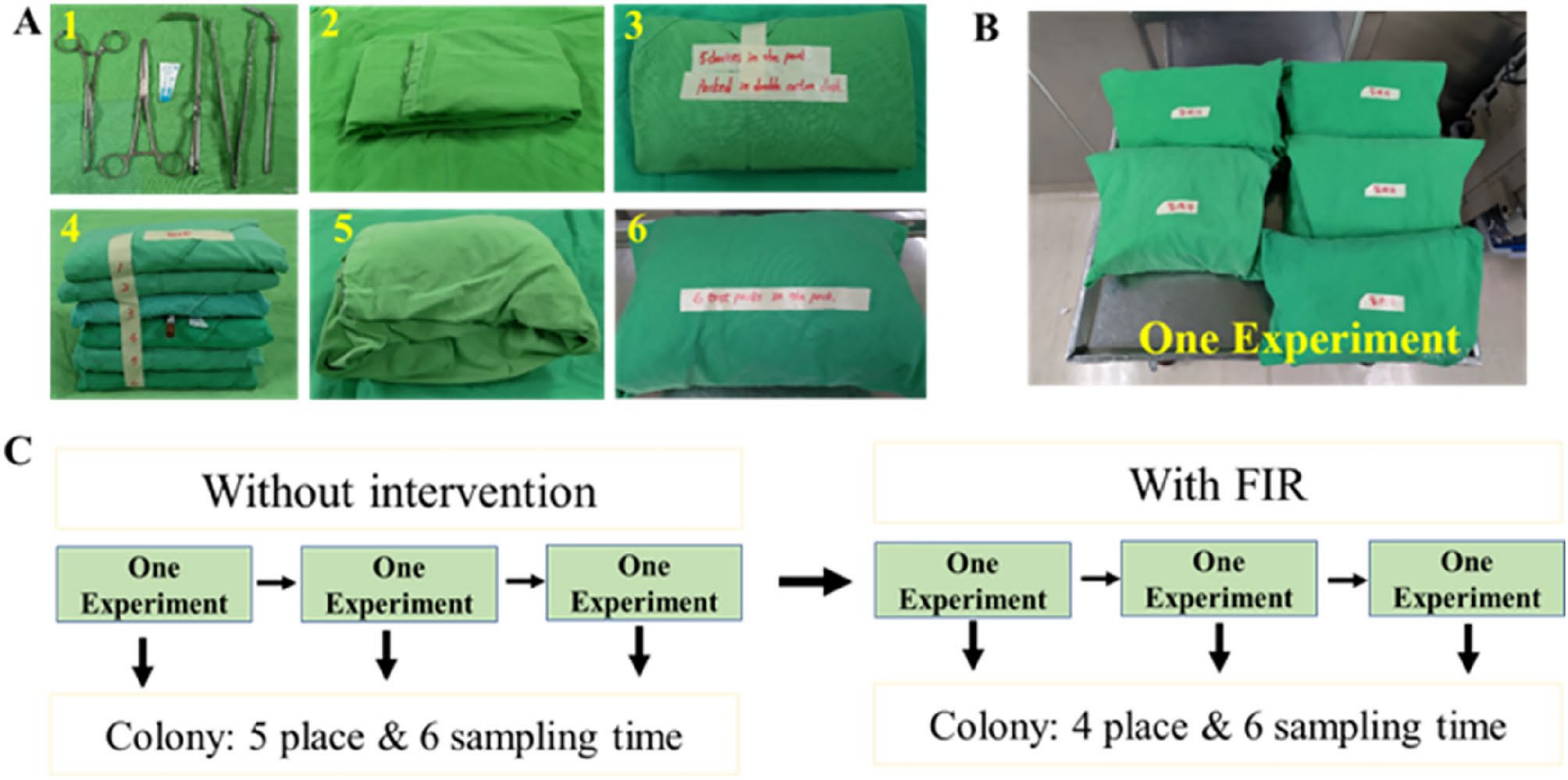
Flow diagram of the study. (**A**)There are five packages (for five monitoring points) and six test packages (six sampling times) within one set of the experiment. (**C**) Region and sampling time of colony culture in this experiment.

This study was conducted in the OR of Ren'ai District, Taipei City Hospital, from September 2021 to July 2022. After sterilisation of the surgical sets, the sterile instrument kits were stored in a room purified with high-efficiency particulate air filter. The nursing staff prepared a surgical instrument packaging using double-layer cotton cloth using the parcel-wrap technique; the package contained five instruments and a strip-type biological sterilisation validation indicator (Browne Class 6 Prevac TST Integrator) to verify the sterilisation process. The surgical instrument package was steam-sterilised at 134 °C for 3 min and 30 s in the same autoclave to complete the sterilisation process. Five storage areas (neurosurgery [NS], obstetrics and gynaecology [OBGYN], otorhinolaryngology [ENT], gastrointestinal surgery [GIS], and orthopaedics [ORT]) were used as sampling points, and six bags were placed in each storage area. Bacterial sampling was performed on days 0, 3, 6, 12, 24, and 30, during which time the temperature and relative humidity were recorded. After the first phase of the experiment was repeated three times, in the second phase, four storage areas (NS, OBGYN, ENT, and ORT) were treated with FIR, and the experiment was repeated three times (Fig. 2).

### Sampling methods

**Figure 3:**
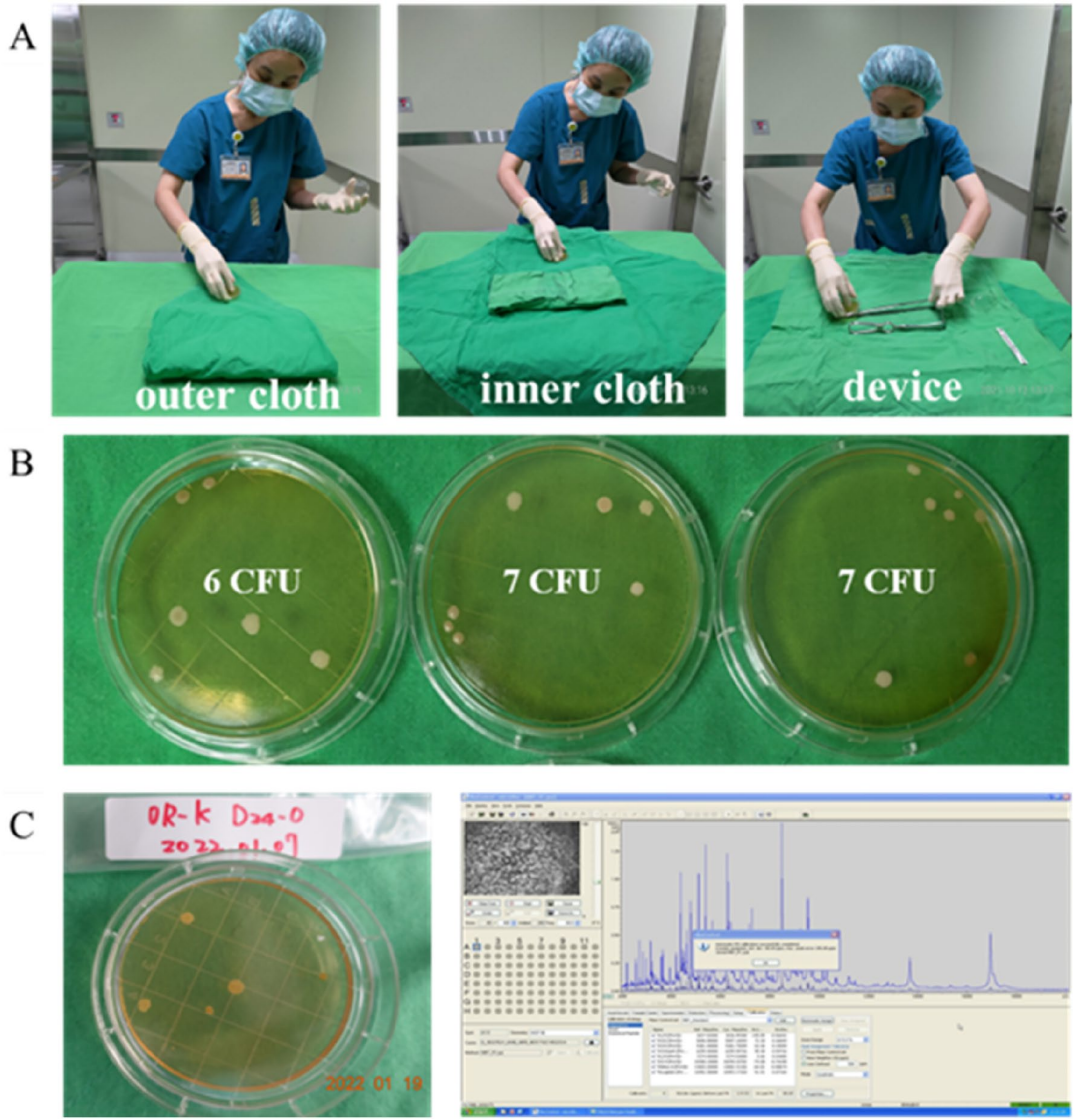
Stamp-form contact-plate. (**A**) Sampling technique. (**B**) Colony forming units counted with unaided eye after incubation at 35℃ for 48 h and then 120 h in room; the average of all counts is 20/3 = 6.67 CFU/plate. (**C**) Bacterial identification through matrix-assisted laser desorption/ionisation time-of-flight mass-spectrometry analysis (MALDI Biotyper smart, Bruker, Germany).

We used stamp-form contact plates containing Sabouraud dextrose agar and tryptic soy agar which were cultured for fungi and bacteria, respectively. (Dr. Plate Biotech Company, Taiwan, Supplementary Fig. 2). The agar plate was placed in contact with the sample through an aseptic technique in a clockwise direction at an interval of 3–5 cm for 2 s, and the sample was obtained separately from the outer cloth, inner cloth, and devices in sequence. After one-step sampling and inoculation, the samples were placed in zipper bags and transferred to the hospital microbiology laboratory. After culturing in a 35 °C incubator for 48 h, they were set to stand at room temperature (laboratory temperature 25–27 °C) for 5 days. The number of colonies on the agar plates was recorded visually (Fig. 3).

### Bacterial identification

We used a high-throughput microbial identification system-MALDI Biotyper smart, Bruker, Germany (matrix-assisted laser desorption/ionisation-time of flight mass spectrometry) for mass detection and analysis of the structural proteins of the bacteria itself; the detection mass range was 2000–20,000 Da, the colonies were first cultured for 24–48 h, and the colonies to be tested were mixed with matrix reagents (matrix) and uniformly smeared on the sample plate to form a protein-matrix crystalline film. After the sample plate was placed in the instrument, excitation with laser light in a vacuum state causes the peptide molecules (peptide) to desorb from the protein sample and accept the positive charge provided by the matrix (matrix) to become charged ions. After being accelerated by the electrodes, charged ions enter the vacuum flight tube and reach the ion detector at the end of the vacuum tube according to their mass/charge ratio. The ion detector recorded the flight time and signal intensity of ions of different masses and translated them into mass/charge ratio maps. The computer system automatically compared the similarity between detected sample maps of known strains in the database and confirmed the strain identity. The identification of the microorganism is completed after the process, and the type of colony is consistent. If it cannot be confirmed, identification is performed by repeated staining.

### Statistical analyses

Continuous variables are presented as mean and standard deviation, and differences in characteristics between groups were analysed using Student’s t-test, one-way analysis of variance (ANOVA), and Scheffe’s post hoc test. Categorical variables are presented as numbers and percentages. Binary and ordinal generalised estimating equation (GEE) methods, an extension of generalised linear models that allow for the analysis of repeated measurements, were used to investigate the association between CFU count and FIR. Sampling time, location, temperature, and humidity were adjusted in the GEE models to demonstrate the independent association between CFU count and the risk of FIR. Statistical analysis was performed using the SPSS software version 20 (IBM Corp., Armonk, NY, USA). Statistical significance was set at p < 0.05.

## Results

**Figure 4:**
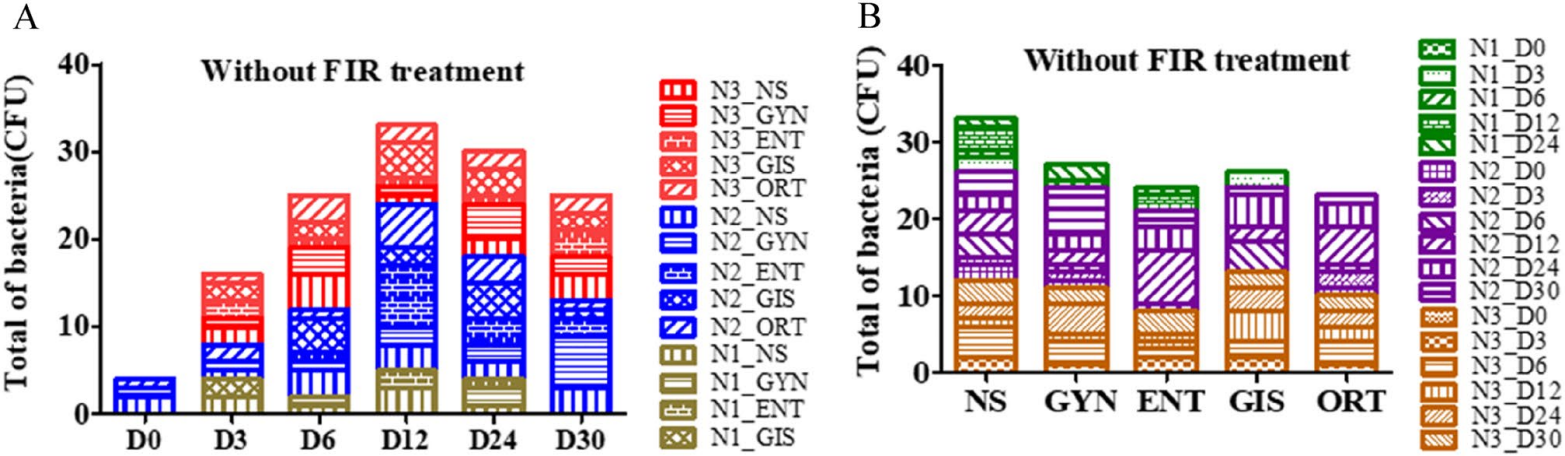
Distribution of 58 packages with colonies (**A**) by time and (**B**) by location of storage area.

In the first stage, samples were obtained from 85 surgical instrument packages without FIR treatment. Colonies were observed in samples from the outer layer of cloth in 58 packages (68.2%), and no colonies were observed in samples from the inner layer of cloth and instruments (Table 1). The humidity of the room where the surgical instrument package was stored was 58.70% ± 1.71%, and the temperature was 21.74 °C ± 0.41 °C. In the second stage, 72 surgical instrument packages were treated with FIR. No colonies were observed on any of the samples. Humidity under the heated FIR equipment was 51.39% ± 3.09%, and temperature was 23.13 °C ± 0.59 °C. Without the heated FIR, the humidity below the device was 56.94% ± 4.33%, and the temperature was 20.87 °C ± 0.62 °C (Table 2). The distribution of colonies in the 58 packages is shown in Fig. 4. Three of the 15 samples on day 0 and 100% of the samples on day 30 had colonies; 60% of the samples in each of the five storage areas had colonies (Table 3).

**Table 1:** Results of samples with microbial growth in all autoclaved packages in the study.

	Without FIR			Heated FIR		Non-heated FIR	
	N = 85			N = 54		N = 18	
	Outer cloth	Inner cloth	Device	Outer cloth	Device	Outer cloth	Device
Microbial growth	58 (68.2%)	Not detected	Not detected	Not detected	Not detected	Not detected	Not detected
No growth	27 (31.8%)	85	85	54	54	18	18

Data with categorical variables are reported using number (percentages).

*FIR* far-infrared radiation.

**Table 2:** Temperature and humidity used in the study.

	N	Temperature, ℃		Humidity, %	
		Mean	Std. Dev	Mean	Std. Dev
Without FIR	157	21.74	0.41	58.70	1.71
Heated FIR	54	23.13	0.59	51.39	3.09
Non-heated FIR	18	20.87	0.62	56.94	4.33

Continuous variables are presented as mean and standard deviation.

**Table 3:** Distribution of 58 of the 85 packages with colonies by time and place of storage areas.

	N	Count	%		N	Count	%
Sampling time				Place			
D0	15	3	20	NS	17	14	82
D3	15	8	53	GYN	17	12	71
D6	15	12	80	ENT	17	11	65
D12	15	12	80	GIS	17	11	65
D24	15	13	87	ORT	17	10	59
D30	10	10	100				
Total	85	58	68		85	58	68

Data with categorical variables are reported using numbers (percentages).

The two groups with and without FIR treatment showed significant differences in the CFU in the outer packaging on days 3, 6, 12, 24, and 30 using an independent sample t-test (Table 4). The statistical analysis of samples (ANOVA) is presented in Table 5, which includes the parameters of sampling time and location of storage. According to the obtained F, the significance of results (F = 5.719) for the alpha level selected (0.05) was evident, indicating a significant difference in the CFU on the 6 days of sampling. Scheffe’s method for simultaneous joint pairwise comparison shows significant difference on day 6. According to the obtained F, the parameters of the storage place were not the most significant of the results (F = 0.419, p = 0.794).

**Table 4:** Comparison of colony-forming units between intervention with FIR and without FIR by sampling time.

	Without FIR		With FIR	
	Mean	Std. Dev	Mean	Std. Dev
Sampling time				
D0	0.33	0.65	Not detected	Not detected
D3*	0.67	0.89	Not detected	Not detected
D6**	1.58	1.51	Not detected	Not detected
D12**	2.17	2.13	Not detected	Not detected
D24***	2.00	1.35	Not detected	Not detected
D30***	2.50	1.43	Not detected	Not detected

*, **, and *** indicate significant differences between without FIR and with FIR treatment groups; p < 0.05, < 0.01, and < 0.001, respectively.

**Table 5:** Colony-forming units (CFU) of the 58 packages with colonies by time and place of storage areas.

	N	Mean	Std. Dev	F
Sampling time				5.719***
D0	3	0.27	0.59	
D3	8	0.87	0.92	
D6	12	1.67	1.40	
D12	12	2.20	1.97	
D24	13	2.00	1.25	
D30	10	2.50	1.43	
**Scheffe’s post hoc : D30 = D12 = D24 = D6 > D3 = D0**				
Place				0.419
NS	14	2.29	0.91	
GYN	12	2.17	1.53	
ENT	11	2.18	1.78	
GIS	11	2.45	1.13	
ORT	10	2.10	1.29	

One-way ANOVA and Scheffe’s post hoc test revealed significant differences in the number of CFU from various sampling times and places. ***p < 0.001.

GEE was used to examine the interaction of sampling time (days 0, 3, 6, 12, 24, and 30), storage location (classified as NS, OBGYN, ENT, GIS, and ORT), temperature, and humidity. The generalised linear model was extended to obtain correlations between the adjusted observations. The analysis results showed that after controlling for the sampling time, storage location, temperature, and humidity, the adjusted relative risk of FIR treatment was 0.0179 and 0.194 compared with the no FIR treatment, and there was a significant difference (p < 0.001), indicating that the probability of colony formation after FIR treatment is low (Table 6).

**Table 6:** Risk factors associated with colony-forming units (CFU) in a generalised estimating equation (GEE) model.

	Adjusted RR	95% CI	95% CI	p
Intervention				
Without FIR	Reference			
Heated FIR	0.179	0.107	0.299	***
Non-heated FIR	0.194	0.078	0.487	***
Sampling time				
D0	Reference			
D3	1.512	1.124	2.034	**
D6	2.367	1.668	3.359	***
D12	3.190	1.989	5.117	***
D24	2.560	1.803	3.635	***
D30	3.487	2.296	5.297	***
Place				
NS	Reference			
GYN	0.843	0.390	1.825	
ENT	0.798	0.584	1.089	
GIS	0.905	0.539	1.520	
ORT	0.735	0.587	0.920	**
Temperature	0.644	0.343	1.211	
Humidity	0.910	0.794	1.042	

GEE modelling is used to account for multiple measurements per CFU, while adjusting for intervention, sampling time, place, temperature, and humidity. RR, relative risk; CI, confidence interval. *, ** and *** indicate significant differences with reference; p < 0.05, < 0.01, and < 0.001, respectively.

Microbial distribution in the 58 packages with colonies is shown in Table 7. The total number of colonies was 130 CFU, and 34 bacterial species were identified. The main species were *Staphylococcus* (35%), *Bacillus* (21%), *Kocuria marina* and *Lactobacillus* (14%), and mould (5%).

**Table 7:** Microbial distribution of the 58 packages with colonies.

Organisms	Number	%	Organisms	Number	%
*Agromyces subbeticus*	1	0.8%	*Pseudomonas lundensis*	1	0.8%
*Arthrobacter luteus*	2	1.5%	*Pseudomonas oryzihabitans*	2	1.5%
*Arthrobacter russicus*	1	0.8%	*Pseudomonas vancouverensis*	1	0.8%
*Bacillus* spp.	27	20.8%	*Roseomonas mucosa*	2	1.5%
*Deinococcus species*	2	1.5%	*Sphingomonas desiccabilis*	1	0.8%
*Dermacoccus nishinomiyaensis*	1	0.8%	*Trichosporon*	1	0.8%
*Enterobacter cloacae*	3	2.3%	*Staphylococcus*		
*Gordonia* spp.	3	2.3%	*Staphylococcus aureus*	3	2.3%
*Kocuria marina*	9	6.9%	*Staphylococcus capitis*	11	8.5%
*Lactobacillus*	9	6.9%	*Staphylococcus carnosus*	1	0.8%
*Massilia timonae*	1	0.8%	*Staphylococcus cohnii*	4	3.1%
*Microbacterium species*	3	2.3%	*Staphylococcus epidermidis*	4	3.1%
*Mould*	7	5.4%	*Staphylococcus haemolyticus*	2	1.5%
*Paenibacillus species*	1	0.8%	*Staphylococcus hominis*	15	11.5%
*Paracoccus yeei*	2	1.5%	*Staphylococcus pettenkoferi*	1	0.8%
*Penicillium* spp.	2	1.5%	*Staphylococcus saprophyticus*	3	2.3%
*Proteus mirabilis*	1	0.8%	*Staphylococcus wameri*	2	1.5%
*Providencia rettgeri*	1	0.8%			
Total	130	100%			

Data with categorical variables are reported using numbers (percentages).

## Discussion

In this study, we found that FIR treatment of surgical instrument packages is effective in reducing the growth of microorganisms and may be useful in reducing the incidence of surgical-site infections from transfer of contaminants from surgical packages during operations.

Sterile condition is a necessity during surgery. It is important to ensure that instruments are sterile before surgery and that contaminants are not transmitted to the surgical site through the packaging material of surgical instruments. Therefore, maintaining the shelf life of instruments after sterilisation is an effective strategy to ensure maximum protection against potential contamination and surgical-site infection. We determined that FIR can inhibit the growth of various environmental microorganisms and reduce bacterial contamination of the outer packaging of stored surgical trays, while also extending the sterile shelf life. It also reduces the cost of resources and time needed for the re-sterilisation process.

The more people in the OR, the more bacteria in the air^34^; air quality also depends on the number of bacteria expelled by the OR personnel^35,36^. The movement of the staff generates airborne particles, especially 0.3–0.5-μm particles, from the floor, hem of shoes, and clothing^37^. The results of a study investigating bacterial air contamination in covered and uncovered sterile areas of the OR over 24 h showed that, in the uncovered group, the number of CFU at different time points ranged from 2 to 30, with an estimated mean survival time of 2.8 h, and a correlation between time and bacterial contamination was noted^38^. The study isolated 16 different microorganisms, including *Cutibacterium acnes*, *Micrococcus luteus*,* S. epidermidis*,* S. warneri*,* S. hominis*,* S. capitis*,* M. flavus*, *Brachybacterium* sp.,* S. saprophyticus*,* S. simulans*,* S. haemolyticus*, *Micrococcus* sp.,* B. cereus*, *Ewingella americana*, *Corynebacterium* sp., gram-positive rods, and unidentified; the total number of CFU was 98. This is similar to our results in which microorganisms from the outer packaging of sterile instruments without FIR treatment were cultured for 3 days. We found 34 microbial species in 58 samples in 30 days, one-third of which belonged to microbes found on the surface of the human body, and one-third were environmental bacteria.

From a clinical standpoint, airborne bacteria can contaminate wounds during surgery, and past research has shown that air pollution is indeed a problem^39–41^. There is no indicator of the duration for which sterile items can be considered uncontaminated before surgery. In this study, we compared device trays, double-layer cotton wrapping, and sterilisation processes, and stored them with or without FIR treatment. We speculated that the number of microbes on the outer cloth used to wrap the device can be reduced. As expected, FIR treatment protected the outer packaging of the device from contamination. Culture samples from the 72 items with FIR treatment were negative, and the probability of a positive result for at least 30 days was 0. Similar results were obtained in a microbial contamination study of cumin seeds using FIR treatment^42^. FIR may have implications for the maintenance of sterile areas in the storage environment.

The storage area must be restricted and away from windows or traffic. It is essential to store sterile packages in a dry, well-ventilated area with open racks for air circulation and moderate temperatures and humidity; storage in closed cupboards in busy clinical areas must be avoided, and stock packages should be stored separately^7^. Microbial culture in items that did not receive FIR treatment confirms that the device package is almost always contaminated with various microorganisms, thus posing a challenge to the spread of microorganisms in ambient air. However, no viable microorganisms were detected in almost 100% of the cultures, confirming the bacteriostatic efficiency of the FIR treatment. Further, there may be a 6%–12% drop in the humidity of the environment in the storage area, which is not conducive to the living conditions of the microorganisms, although factors of environmental humidity and temperature did not reach statistically significant differences. In conclusion, the use of the FIR equipment significantly reduces the risk of bacterial contamination in the outer packaging of sterile items and may considerably save labour (repacking time) and re-sterilisation costs by extending the shelf life.

## Conclusions

This study demonstrated that FIR treatment can be used as an easy and safe method for the decontamination of sterile instrument storage spaces in the OR. FIR treatment reduces the number of microorganisms on the outer packaging of sterilised instruments to 0 and does not increase the workload of the OR personnel. Therefore, the safety and quality attributes of the surgical site are better maintained.

## Supplementary Information

Supplementary Information 1.

Supplementary Information 2.

Supplementary Information 3.

## Data Availability

The data that support the findings of this study are available from the operating room of Ren'ai District, Taipei City Hospital, but restrictions apply to the availability of these data, which were used under license for the current study, and so are not publicly available. Data are however available from the authors upon reasonable request and with permission of the operating room of Ren'ai District, Taipei City Hospital.
