# Supplementary material for: Effect of far-infrared radiation on inhibition of colonies on packaging during storage of sterilised surgical instruments
Source: Sci Rep. 2023 May 25;13:8490. doi: 10.1038/s41598-023-35352-9 (PMC10212960; doi:10.1038/s41598-023-35352-9)
Supplement: Supplementary file 2 — Supplementary Information 2. [file 41598_2023_35352_MOESM2_ESM.pdf]

TEST REPORT TUCHENG

COPY

Date of Issue: Feb.24,2022 Date of Receipt and Test Start: Feb.23,2022

Report No.: TFF1B252 Quantity: 1PC Page Order/Pages: (P2/2) Ref. No.: NIL

Report Title: National Defense Medical Center(M0127) Item: Product

Address: No. 161, Sec. 6, Minquan E. Rd., Neihu Dist., Taipei City 114 , Taiwan (R.O.C.)

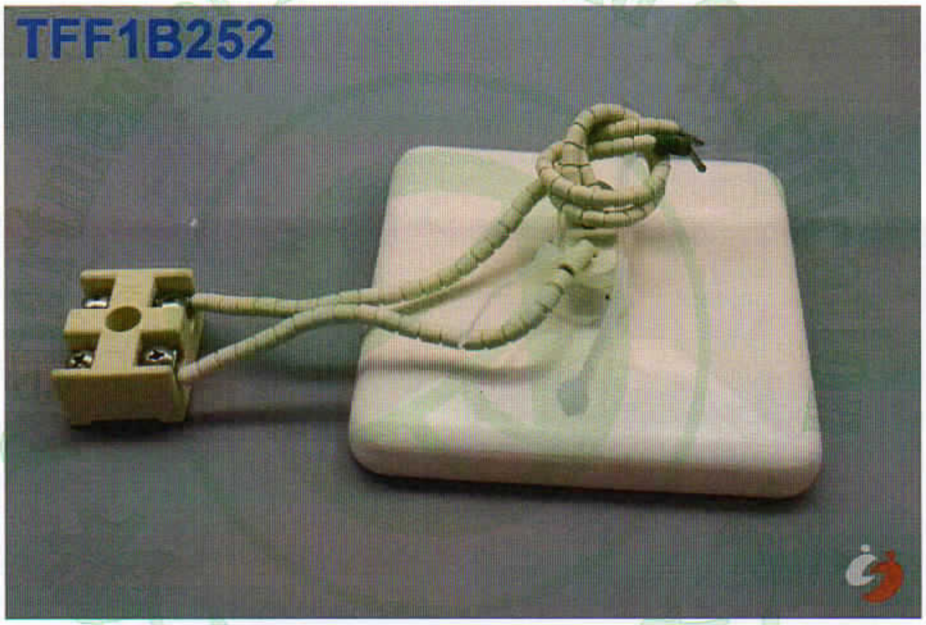

Note: 1.This report is only responsible for the submitted sample(s), which will be kept for one month period.

2.This report cannot be reproduced in any way, except in full context, without the prior approval in writing of this Department of Testing and Certification.

3.The test report should not be used for public advertisement and commercial promotion.

Authorized by president of Taiwan Textile Research Institute Jui-hung Kao

Director,
Department of Testing and Certification

Department of Testing and Certification, Taiwan Textile Research Institute
No.6, Chengtian Rd., Tucheng Dist., New Taipei City 23674, Taiwan (R.O.C.)
Tel : +886-2-22670321 ext. 7107, 7110
Fax : +886-2-22675108 , +886-2-22689839
