# Supplementary material for: Effect of far-infrared radiation on inhibition of colonies on packaging during storage of sterilised surgical instruments
Source: Sci Rep. 2023 May 25;13:8490. doi: 10.1038/s41598-023-35352-9 (PMC10212960; doi:10.1038/s41598-023-35352-9)
Supplement: Supplementary file 3 — Supplementary Information 3. [file 41598_2023_35352_MOESM3_ESM.pdf]

Certificate of Analysis

Product Name : Sabouraud Dextrose Agar Contact
Cat. No. BDPS010C
Lot number : 111011803
Manufacturing date : 2022.01.18
Trace code : AGOA-5
Expiry date : 2022.05.18

(1) Appearance

**Color of Agar**	Transparent Yellow
**Bubble formation**	No
**pH Value**	5.7 (Expected final pH: 5.6 ± 0.2 @25°C)

(2) Microbiology Test

**Microorganisms**	**Result**
Aspergillus brasiliensis (ATCC 16404)	Growth
Candida albicans (ATCC 10231)	Growth

*Incubate at 22.5±2.5°C incubation for 3-5 days.
ATCC is a registered trademark of the American Type Culture Collection, Manassas, VA 20108, USA.

(3) Sterile Test

No colonies formation after 3 days incubation at 32.5±2.5°C incubator and 5 days incubation at 22.5±2.°C incubator, which defined as non-contaminated agar.

Laboratory Results: Validation

Testing performed by: ZACK

Director of Technical Services: [signature]

品管部
2022.1.24

達特普雷生技股份有限公司
台北市內湖區新明路143巷8號1樓
Tel:(02)2792-8776 Fax:(02)2793-8981
www.drplatebiotechnology.com
